# Single Cell RNA Sequencing of Papillary Cancer Mesenchymal Stem/Stromal Cells Reveals a Transcriptional Profile That Supports a Role for These Cells in Cancer Progression

**DOI:** 10.3390/ijms26104957

**Published:** 2025-05-21

**Authors:** Danny Jandu, Nani Latar, Artida Bajrami, Rachel Queen, Megan Hasoon, Matthew Teasdale, Rafiqul Hussain, Jonathan Coxhead, Sebastian Aspinall, Annette Meeson

**Affiliations:** 1 Bioscience Institute, Newcastle University, International Centre for Life, Newcastle upon Tyne NE1 3BZ, UK; dannyjandu@hotmail.co.uk (D.J.);; 2 Department of Surgery, Faculty of Medicine, Universiti Kebangsaan Malaysia Medical Centre, Cheras, Kuala Lumpur 56000, Malaysia; 3 Computational Biology Facility, Liverpool Shared Research Facilities, Faculty of Health and Life Sciences, University of Liverpool, Liverpool L69 3DR, UK; 4 Bioinformatics Support Unit, The Faculty of Medical Sciences, Newcastle University, Newcastle upon Tyne NE2 4HH, UK; 5 Department of General Surgery, Aberdeen Royal Infirmary, Aberdeen AB25 2ZN, UK; sebastian.aspinall@nhs.scot

**Keywords:** mesenchymal stem/stromal cells, papillary thyroid cancer, normal thyroid, single cell RNA sequencing

## Abstract

Papillary thyroid cancer (PTC) contains mesenchymal stem/stromal cells (MSCs), but their contribution to PTC progression is not clear. In this study, we compared the transcriptional signatures of normal thyroid (NT) and PTC-derived MSCs with the aim of determining if these have distinct transcriptomes that might influence PTC progression. We used flow cytometry in combination with a panel of MSC clusters of differentiation (CD) markers and showed that both thyroid MSC populations expressed MSC markers and lacked expression of markers not normally expressed by MSCs. In addition, we determined that both MSC populations could differentiate to adipocytes and osteocytes. Analysis of single cell RNA sequencing data from both MSC populations revealed, regardless of tissue of origin, that both contained similar numbers of subpopulations. Cluster analysis revealed similarity in expression of both MSC populations for stromal markers, the vascular marker *VEGFA* and the smooth muscle marker *CALD1*, while smaller subpopulations expressed markers of more lineage-committed thyroid cells. PTC MSCs also showed upregulated expression of 28 genes, many of which are known to be involved in epithelial–mesenchymal transition (EMT) and/or disease progression in several types of cancers, including but not limited to breast cancer, gastric cancer, cervical carcinoma, bladder cancer and thyroid cancer. This included several members of the *S100* and *IGFBP* gene families. Taken together, these data support a role for PTC MSCs in PTC progression.

## 1. Introduction

Studies of Mesenchymal stem cells (MSCs) in cancer suggest that these cells are tumor suppressive in some cancers, while in others they promote tumor growth [1]. Epithelial-to-mesenchymal transition (EMT) is an important process in cancer progression in many cancers and involves changes to epithelial cell characteristics, leading to a mesenchymal cell phenotype that is more migratory and invasive. However, it is now recognized that during the EMT process, many cells can exist in hybrid states, having characteristics of both cell types. Cells with these characteristics are considered to have undergone partial EMT, which can be reversed [2].

There is evidence that MSCs can induce EMT; for example, when bone marrow (BM) MSCs were co-cultured with cells of the breast cancer cell lines MDA-MB-231, T47D and Sk-Br3, the cancer cells showed increased expression of EMT markers and increased expression of genes associated with invasion and angiogenesis [3]. Meanwhile, direct contact co-cultures of BM MSCs with KM12SM colon cancer cells also resulted in increased migration, proliferation and increased expression of EMT markers by the cancer cells [4].

Currently, there are only a small number of publications on MSCs in papillary thyroid cancer (PTC), and the role of these cells in promoting PTC remains unclear. In 2017, MSCs were isolated from both adult normal thyroid (NT) tissue and PTC. Both populations of cells were plastic adherent, and their phenotype was comparable to that of BM MSCs [5]. Moreover, the isolated PTC MSCs were reported to express higher levels of genes related to desmoplasia compared to normal MSCs. This difference in fibrotic gene marker expression suggests that these cell populations were in different differentiation stages in relation to their functional states, with higher expression of fibrotic gene markers in PTC MSCs being associated with a more proliferative and invasive behavior [5].

In this study, we hypothesized that there would be differential gene expression between PTC and NT MSCs that would support a role for PTC MSCs as important drivers for PTC progression, possibly via the EMT process. We isolated MSCs from both NT tissue and PTC and have shown that a number of differentially expressed genes are upregulated in the PTC MSCs that have been reported to be important in EMT, stem cell and/or cancer stem cell regulation and cancer progression.

## 2. Results

### 2.1. Cells with Mesenchymal Stem Cell Characteristics Can Be Isolated from Normal Thyroid Tissue and Papillary Thyroid Cancers

**Figure 1:**
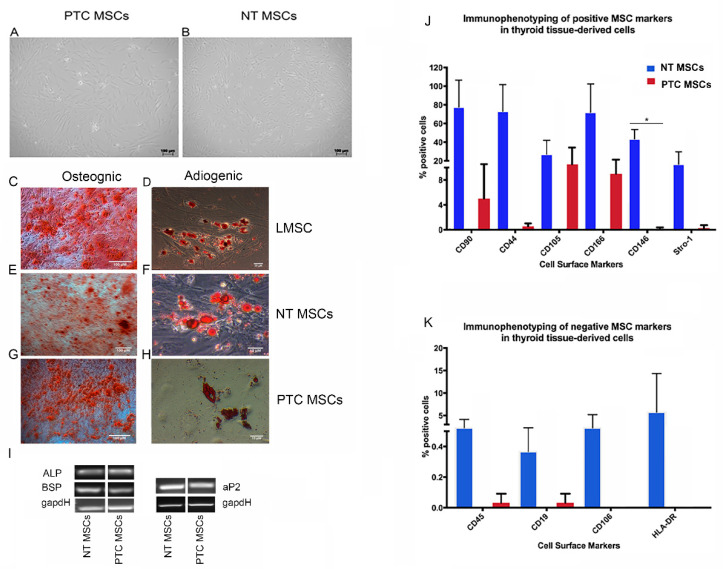
Analysis of mesenchymal stromal cells derived from normal thyroid and papillary thyroid cancer. Representative images were taken from three biological replicates for each tissue type, *n* = 3. Brightfield images show the characteristic MSC morphology displayed by NT MSCs (**A**) and PTC MSCs (**B**). Scale bars = 100 µM. Results of histological analysis are shown; note that LMSCs were used as a positive control for both osteogenic (**C**) and adipogenic differentiation (**D**). Both thyroid MSC populations differentiated to the osteogenic and adipogenic lineages as shown for NT MSCs (**E**,**F**) and for the PTC MSCs (**G**,**H**). Note that Alizarin red staining of all three MSC populations, indicating matrix mineralization, occurred, indicating successful osteogenesis (**C**,**E**,**G**) and Oil Red O staining of lipid vesicles within all cell populations differentiated to adipocytes (**D**,**F**,**H**). Further confirmation of differentiation to osteogenic and adipogenic lineages by both thyroid MSC populations was shown using semi-quantitative PCR to confirm mRNA expression of genes associated with these differentiations; these included for osteogenesis *ALP* (187 bp) and *BSP* (161 bp) and for adipogenic differentiation *aP2* (130 bp). *GapdH* was used as a loading control (150 bp) (**I**). Graphical representation of immunophenotyping results for both thyroid cell populations showing expression of known MSC markers (**J**) and for expression of markers normally expressed at low/no levels by MSCs (**K**). Note the significant difference in expression of CD146 in the NT MSCs above that of the PTC MSCs * *p* = <0.05.

Cells from both tissue types were plastic adherent and showed typical MSC morphology under standard MSC culture conditions, (Figure 1A,B). In addition, we examined all cell populations for the ability to differentiate to the osteogenic and adipogenic lineages. Limbal MSCs (LMSCs) were used as a control. All cell populations were capable of both osteogenic differentiation, as indicated by the Alizarin Red staining for matrix mineralisation (Figure 1C,E,G), and adipogenic differentiation, as indicated by the staining of lipid vesicle in cells with Oil Red O (Figure 1D,F,H).

Additionally, we undertook semi-quantitative PCR analysis to confirm osteogenic differentiation and showed that both thyroid MSCs populations expressed alkaline phosphate (*ALP*) and bone sialoprotein 1 (*BSP*) (Figure 1G). To confirm adipogenic differentiation, we showed that both thyroid MSC populations expressed adipocyte binding protein 2 (*aP2*) (Figure 1I). In both cases, GapDH was used as a loading control.

PTC MSCs and NT MSCs were immunophenotyped at passage three. Both populations expressed markers of MSCs. In all cases, the expression of MSC markers was higher in NT MSCs than PTC MSCs, but this difference was only statistically significant for expression of CD146 (Figure 1J).

In addition, the cells were immunophenotyped for expression of markers normally absent or expressed at low level in MSCs; these included the hematopoietic lineage markers CD45, CD19 and CD106, which have been reported to be expressed by only a subpopulation of MSCs, and HLA-DR, a marker for human major histocompatibility complex HLA-DR (MHC II). All cell populations expressed low levels of CD45 and CD19, but only NT MSCs expressed low levels of CD106 and HLA-DR, while PTC MSCs did not express either of these two markers (Figure 1K).

### 2.2. Single Cell RNAseq Cluster Analysis for Select Genes Used to Annotate the Subpopulations Within Both MSC Populations

**Figure 2:**
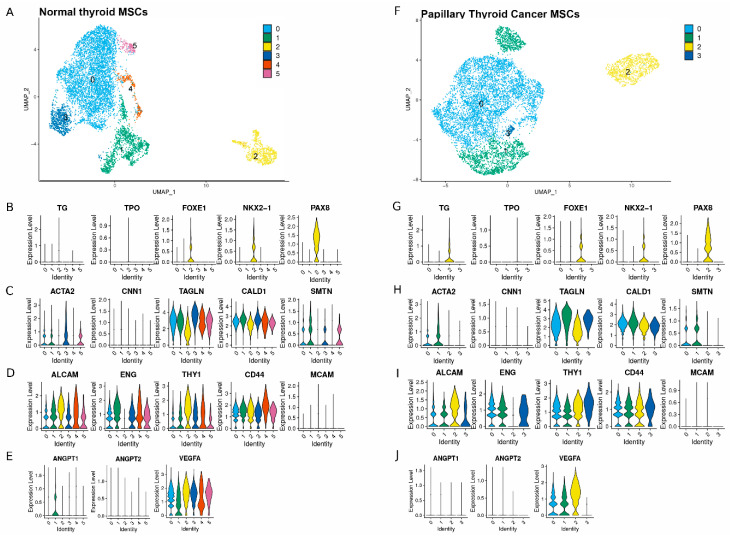
Annotation of the MSCs subpopulations using integrated single cell RNA sequencing. (**A**) Identification of MSC clusters for NT MSCs (**F**) and for PTC MSCs. *n* = 2 patients MSCs for both tissue types were used for clustering analysis; profiles are shown of combined data from 2 cultures of each tissue type. UMAPs represent a clustering analysis of 8000 cells at a resolution of 0.1; this resulted in 6 clusters in the MSCs from NT and 4 from PTC (**A**,**F**). Violin plots show NT MSCs (**B**) and PTC MSCs (**G**) expressing thyroid lineage-committed cell markers, NT MSCs (**C**) and PTC MSCs (**H**) expressing smooth muscle markers, NT MSCs (**D**) and PTC MSCs (**I**) expressing stromal markers and NT MSCs (**E**) and PTC MSCs (**J**) expressing angiogenic markers.

Clustering analysis of 8000 cells at a resolution of 0.1 resulted in identification of six clusters in the NT MSCs and four in the PTC MSCs (based on pooling cells isolated from 2 NT and 2 PTC donors). (Figure 2A,F). Cells expressing genes associated with thyroid lineage-committed markers were found to be primarily located in cluster 2 in the NT MSCs and the PTC MSCs (Figure 2B,G). We observed low expression of thyroid peroxidase (*TPO*) in cluster 2 for both cell populations. However, there was higher expression of *FOXE1*, *NKX2-1* and *PAX8* in cluster 2 in both the NT and PTC MSCs compared to the other clusters (Figure 2B,G), with *PAX8* being the most robustly expressed marker. We also observed more PTC MSCs expressed thyroglobulin (*TG*) in cluster 2 than in any other cluster, and this was more widely expressed than that of NT MSCs in cluster 2.

Both cell populations were also examined for distribution of expression of smooth muscle markers (Figure 2C,H). The most robustly expressed markers were *CALD1* and *TAGLN*, which were expressed in all clusters in both MSC populations, whereas there was much lower expression of *ACTA2* and *SMTH*, with the lowest expression across all clusters for both cell populations being *CNN1*.

In addition, both cell populations were examined for expression of stromal markers *THY1, CD44, MCAM, ALCAM* and *ENG* (Figure 2D,I). Both MSC populations expressed *THY1, CD44*, and *ALCAM* across all clusters. *MCAM* expression was low and was only detected in 2.5% of cells in the NT MSCs and 0.9% of the PTC MSCs (Supplemental Table S1).

We also looked at both cell populations for expression of angiogenic markers (Figure 2E,J) and observed that *VEGFA* was expressed in all clusters for both cell populations, but expression was low in PTC MSC cluster 3. Expression of both *ANGPT1* and *ANGPT2* was low in clusters 0–5 for NT MSCs, except for cluster 1 for *ANGP1*, while expression of *ANGPT1* and *ANGPT2* for all PTC MSC clusters was low or below detection. Further analysis revealed *VEGFA* expression in 88% of NT MSCs and 66% of the PTC MSCs (Supplemental Table S1). Fewer cells of both NT MSCs and PTC MSCs expressed *ANGPT1* (15% for the former and 7% for the latter) and *ANGPT2* (2% for both cell populations, this is summarized in Supplemental Table S1). In addition, we used the data shown as violin plots in Figure 2 and generated UMAPs to show expression of each of these markers across clusters (Supplemental Figure S1).

**Figure 3:**
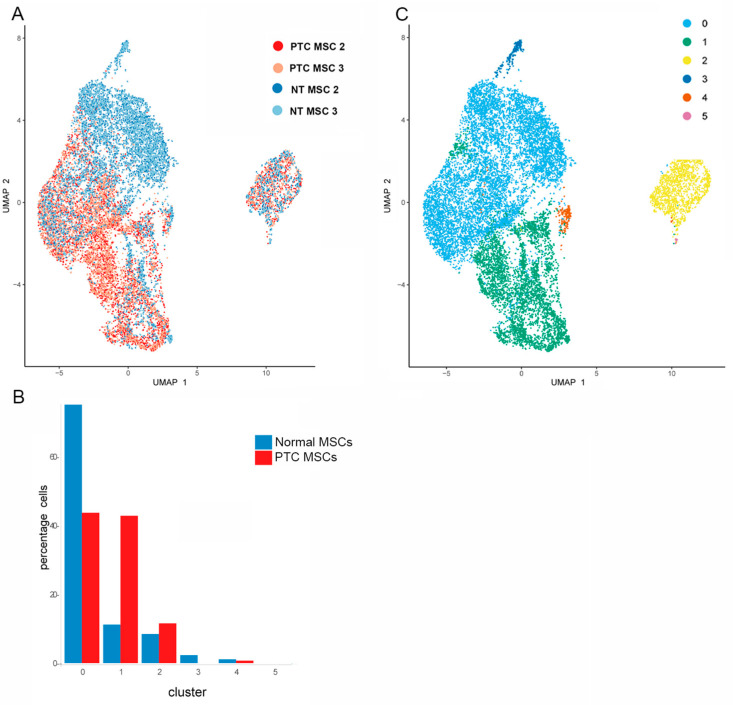
Results of cluster analysis of PTC and NT MSCs. UMAP showing clustering of cells from two NT MSCs cultures and two PTC MSC cultures (**A**). UMAP showing annotation of clusters for both cell types (**C**). Data represent *n* = 2 donors for each tissue type. Graphical representation (using the combined dataset) to determine percentage of PTC and NT MSCs per cluster (**B**). Note the presence of a very small percentage of PTC MSCs in cluster 3, also note the absence of these cells in cluster 5.

To determine how comparable the MSCs were from the same tissue type and how distinct the cells were based on their tissue of origin, we integrated the MSC data from two NT and two PTC MSC donor samples, (Figure 3A). Note that there are some clusters made up of large numbers of NT MSCs for both samples, and some areas showed clustering of large numbers of PTC MSCs from both samples. There are also some areas where the cell types are mixed regardless of tissue of origin. The compositions in terms of percentage of MSCs per cluster are represented graphically (based on the pooled data generated from samples mentioned above) (Figure 3B). Note that cluster 0 contains the highest percentage of both NT and PTC MSCs. A UMAP visualizing the location of the 5 clusters is provided (Figure 3C). As a result of clustering analysis (Figure 3), a distinct cluster has been identified. This cluster contains similar numbers of PTC and NT MSCs (Figure 3B) and has been identified as cluster 2 (Figure 3C). Data on annotation of subpopulations of MSCs (Figure 2) show that this cluster contains cells expressing thyroid lineage commitment genes including *FOXE1, NXK2.5* and *PAX8* for both MSC populations, suggesting this is a subpopulation of cells committed to thyroid lineage differentiation. However, both PT and NT MSCs in this cluster show expression of the stromal markers *CD44, THY1* and *ALCAM* across this cluster (Figure 2D,I, Supplemental Figure S1) suggesting these cells are stromal cells.

### 2.3. Determination of Differentially Expressed Genes Upregulated in PTC MSCs

To determine if transcriptional profiles differed between the NT and PTC MSCs, we looked for differential expression. This analysis revealed higher expression in the PTC MSCs of 28 genes known to be involved in cancer progression (Table 1). This included several members of the *S100A* family and the *IGFBP* family. In addition, all 28 genes were analyzed using cBio Cancer Genomics Portal. Genetic alterations in *WWTR1* impacted negatively on overall survival rates of patients (logrank test *p* value being 0.0230). (Supplemental Figure S2).

### 2.4. Analysis of S100A and IGFBP Gene Family Members Expressed by MSCs

**Figure 4:**
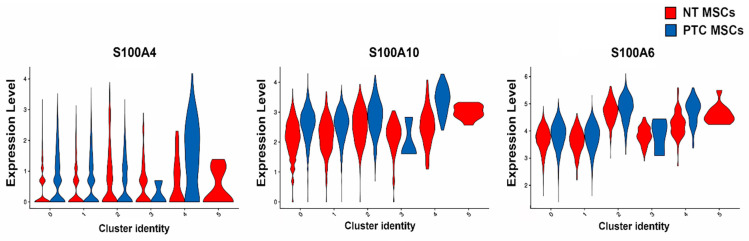
Violin plot representing expression of S100A genes in PTC and NT MSCs per cluster (data used were from two PTC MSC and two NT MSC pooled samples).

**Figure 5:**
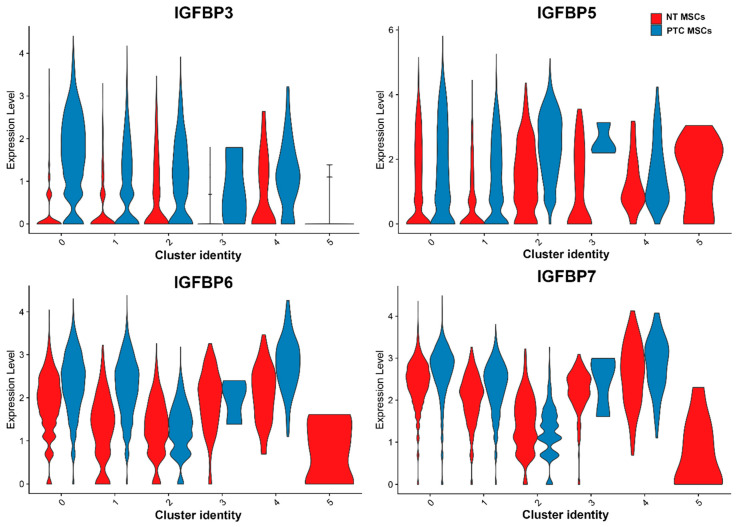
Violin plots representing expression of IGFBP genes in PTC and NT MSCs per cluster (data used were from two PTC MSC and two NT MSC pooled samples).

Expression of genes in cells of different clusters for both PTC and NT MSCs are represented in violin plots (Figure 4 and Figure 5). Cluster analysis showed expression in all clusters (except for cluster 5, which only represents NT MSCs) in both MSC populations of *S100A4, S100A6* and *S100A10*, with expression generally being higher in PTC MSCs than NT MSCs. In cluster 3, there was less expression of *S100A4* and *S100A10* in PTC MSCs (Figure 4). All three genes were differentially expressed having higher expression in PTC MSCs (Table 1).

Several members of the *IGFBP* gene family were also expressed in both cell populations in all clusters (except for cluster 5); this included *IGFPB3, IGFBP5, IGFBP6* and *IGFBP7* (Figure 5). *IGFBP3* was expressed at higher levels in clusters 0–4 in PTC MSCs compared to NT MSCs with expression in the later, being lower in clusters 3 and 5. *IGFBP5* was expressed at higher levels in PTC MSC clusters 0, 1, 2 and 4 compared to NT MSCs, whilst *IGFBP6* was more robustly expressed in PTC MSC clusters 0, 1, and 4 compared to NT MSCs. *IGFBP7* was more highly expressed by PTC MSCs in clusters 0 and 1 compared to NT MSCs. *IGFBP3, IGFBP5, IGFBP6* and *IGFBP7* were all significantly differentially expressed, having higher expression in PTC MSCs (Table 1).

## 3. Discussion

Controversy still surrounds the acronym MSC with regard to its use in denoting mesenchymal stem cell or mesenchymal stromal cell, as indicated by several position papers on this subject by ‘The international Society for Cell and Gene Therapy Mesenchymal Stromal Committee’ in 2005, 2006 and 2019 [6,7,8]. This still appears to be an area of contention with publications still using the acronym MSC for both populations. However, the International Society for Cellular Therapy has defined the minimum criteria for multipotent mesenchymal stromal cells as the following: being capable of proliferation and differentiation; being plastic adherent; expressing CD73, CD90 and CD105; and lacking expression of CD45, CD34, CD14 or CD11b, CD79α or CD19 and HLA class II [6,7]. In 2019, this committee published a statement stating that they still supported the use of the acronym MSC but recommend that this be accompanied by inclusion of tissue source of the cells [8], which we have adhered to in this study. However, we are using MSC to refer to cells in this study as mesenchymal stem/stromal cells, in line with [5].

MSCs have previously been reported to be present in tissue from both non-malignant and PTC sites that have the phenotypic and differentiation characteristics of MSCs [5]. We confirmed that the MSCs we isolated from NT and PTC tissue had MSC characteristics. However, the MSCs in [5] differed to MSCs in our study in that they showed less variation in expression of markers associated with the MSC immunophenotype when compared to each other and with bone marrow MSCs. In this study, we compared expression of MSC markers between PTC and NT MSCs and observed variable expression between PTC and NT MSCs, with expression of several markers being lower in PTC MSCs. However, Parascandolo et al. [5] did not include CD166 or CD146 in their immunophenotyping panel. We included CD146 as it is expressed by a subpopulation of MSCs in bone marrow and associated with a commitment to the vascular smooth muscle lineage [9]. Moreover, CD166 has been reported to be an MSC marker for several MSC populations isolated from a range of tissues [10]. It has been reported that expression of MSC markers may vary depending on the tissue of origin, culture conditions and even disease condition [10,11].

We then undertook cluster analysis of single cell RNA sequencing data for MSCs isolated from different donors. We observed that all donors examined had a similar composition of clusters regardless of tissue of origin of the MSCs. We then generated violin plots using these data to determine the distribution of cells for expression of genes that would enable us to annotate the MSC subpopulations. This revealed that both NT and PTC MSCs had similar patterns of expression of stromal, smooth muscle and angiogenesis markers, and more restricted but similar expression of thyroid lineage-committed cells. Expression of the stromal markers *THY1* (also known as CD90), *CD44, ALCAM* (also known as CD166) and *ENG* (also known as CD105) confirms that our MSCs are of stromal origin. THY1 is not only an MSC marker; it has also been implicated in being important in fate decisions for MSCs, having been reported to be key for osteogenic differentiation and for inhibiting adipogenic differentiation (reviewed in [12]). In addition, CD44 has also been identified as important in the immunoregulation of MSCs [13], and ALCAM is known to be expressed by several MSC populations from a range of tissue sources cultured under different conditions [14].

We also determined whether subpopulations of our MSCs might have angiogenic potential and observed that both MSC populations expressed high levels of *VEGFA* and lower levels of the angiopoietins 1 and 2. MSCs are known to have angiogenic potential. Human colorectal cancer-derived MSCs have previously been reported to enhance angiogenesis in vitro [15]. We also examined the MSCs for expression of smooth muscle markers. MSC populations expressed *CALD1* at similar levels across all clusters. CALD1 was originally considered to be a smooth muscle marker, but it has also been recognized as having a role in the progression of some cancers [16]. The NT MSCs had more clusters showing cells expressing *SMTH, ACTA2* and *CNN1* than the PTC MSCs. MSCs have been previously reported to express some smooth muscle markers; for example, human bone marrow MSCs undergoing myogenic differentiation expressed ATCA2 and CNN1 [17].

We then examined combined scRNA seq data based on two MSC cultures from two donor PTC patients and two cultures derived from NT. We observed differential expression of 28 genes which were more highly expressed in the PTC MSCs than NT MSCs, all of which are known to be involved in cancer progression. These included genes such as SCUBE3, MARCKSL1 and PGK1 in lung cancer [18,19,20], SOX4 and GNAS in breast cancer and hepatocellular carcinoma [21,22,23,24,25], HEG1 in hepatocellular carcinoma [26], PLS3 in pancreatic adenocarcinoma [27], FBN1 and SPARC in gastric cancer [28] and CST1 in gastric cancer and breast cancer [29,30]. EDIL3 has been reported to promote EMT in some breast and prostate cancers [31]. NF2R2 has been implicated in promoting prostate tumorigenesis [32]. LTBP1 has been implicated in promoting esophageal squamous cell carcinoma via EMT [33]. A number of these genes have also been implicated in PTC, for example, WWTR1 [34], CDH6 [35], PRRX1 [36], HIFA [37], S100A4 [38], MAZ [39] and SPOCK1 [40]. SH3BGRL3 mRNA has been reported to have elevated expression in PTC [41] and PCOLCE2 has been reported to be part of a four-gene signature for prediction of prognosis of PTC [42]. S100A10 and S100A6 have been reported to be biomarkers for PTC with lymph node metastasis (LNM) [43]. We also observed differential expression of several members of the *IGFBP* family (Table 1 and Figure 5). IGFBP3 has been reported to be a potential marker for PTC with LNM [44], while IGFBP5 has been reported to be overexpressed in PTC tissue when compared to NT tissue and thyroid multinodular goitres [45]. IGFBP6 has been reported to act as both a tumor suppressor and a promoter of several cancers (reviewed in [46]), but its role in PTC is unknown. However, bone marrow MSCs have been reported to express IGFPB6 [47]. Downregulation of IGFBP7 has been reported to be involved in oncogene-induced senescence in PTC [48], whereas in gastric cancer, IGFBP7 expression has been linked to cancer progression [49]. We observed expression of this gene in both MSC populations. Some MSCs are known to express IGFBP7, expression of which has been shown to be associated with MSC osteogenic differentiation [50], and in some cases in the prevention of cellular senescence [51]. In addition, we examined the TCGA Pan Cancer Atlas study accessed via cBIO Cancer Genomics Portal which found that altered WWTR1 expression was shown to have a negative impact on patient survival rates. This supports our approach as we have yielded information on thyroid MSCs that shows elevated expression of genes in PTC MSCs (including *WWTR1*) that may be important targets to increase patient survival.

This study, however, does have some limitations. These include the small donor sample size and use of scRNA seq, which can include biases in transcript coverage, having a lesser ability to detect low abundance transcripts and, due to the need to isolate cells from tissue, loss of spatial data. The processing of cells into preparations suitable for scRNA seq could also modify the cellular transcriptomics and might impact on cell viability. However, scRNA seq has been used in many cancer studies, as it enables the heterogenous complexity of cells within tumors to be explored, and even enables the identification of rare cell populations including stem cell like cells [52]. Another limitation is the impact of culture, needed for the isolation and expansion of MSCs. It has been reported that long term culture of MSCs can lead to changes at both the phenotypical, functional, and genetic level, many of which are linked to ageing in vitro. Immunophenotypical changes were reported in BM MSCs with increasing passage number, with the most changes being observed in expression of CD106 and CD146 at P7 and P8. In addition, some stemness markers and MSC markers were also downregulated with mRNA expression decreasing by @ 50% at P8 compared to P4 [53]. In our study, to counteract some of these limitations, we have used low passage MSCs and included the use of quality control measures to reduce technical noise and filtered all cells to ensure a consistent quality for inclusion in analysis (see bioinformatics analysis methods). A further limitation is that this study focused on the differences in transcripts between MSCs isolated from well-differentiated PTCs and NTs and has not accounted for any differences in MSCs in variants of PTC, nor does it account for patient demographics. Additionally, functional analysis of the PTC and NT MSCs will be important to determine if the transcriptional signatures identified impact on behavioral abilities of MSCs isolated from PTC and a NT. Future studies will focus on these issues.

## 4. Materials and Methods

### 4.1. Patient Samples

Biopsies for analysis were taken immediately following thyroidectomy from tumors and surrounding normal thyroid glands in adult patients undergoing surgery for well-differentiated thyroid cancer. MSCs from all patients (both PTC and normal) were used for immunophenotyping, osteogenic and adipogenic differentiation and semi–quantitative PCR. MSCs derived from two PTCs and two NTs were used for single cell RNA sequencing.

### 4.2. Isolation of MSCs

Tissue was made available on day of surgery and minced into @2 mm pieces manually; this was then digested in collagenase IV 20 mg/mL (Gibco, Paisley, UK) and DNase 0.01% (Worthington, UK) in 20 mL of complete Roswell Park Memorial Institute 1640 (RPMI-1640) media in a shaking water bath at 37 °C for 90 min. This was then filtered using a 70 µM cells strainer, pelleted and the resulting pellet treated with lysis buffer (23 mL of 0.15 m NH_4_Cl pH 7.5) to remove red blood cells. The cells were then re-pelleted, the lysis buffer was discarded and the cells resuspended and plated in a T25 in complete MSC growth-promoting media (MGPM). This consists of MEM alpha (1X), Gutamax (Gibco, Paisley), 10% FBS (Gibco, Paisley, UK) and 1 mg/100 mL FGF (Peptrotech, London, UK). The media were removed 72 h after seeding cells and the flasks were rinsed with Dulbecco’s phosphate-buffered saline. Fresh MGPM was then added, and the media were changed every 3 days thereafter. All MSCs were cultured at 5% CO_2_ and 5% O_2_. Limbal MSCs were isolated as described previously [54].

### 4.3. Immunophenotyping of MSCs

Immunophenotyping was performed on NT and PTC MSCs at P3. Thyroid MSCs were detached from the tissue culture flask using TrypLE Express and resuspend in FACS buffer to 2 × 10^5^ cells per 100 µL, and labelled with 5 µL of primary antibodies and kept on ice for 60 min. Primary antibodies (purchased from R&D sytems, Abingdon, UK) were CD44, CD90, CD105, CD146, CD166, Stro-1, CD45, CD19, CD106 and HLA-DR. All were primary conjugated with Fluorescein isothiocyanate (FITC) except for HLA-DR. HLA-DR-stained cells were washed post 1 h primary antibody incubation and then stained for 30 min with goat anti mouse IgG FITC-conjugated secondary antibody (Jackson Immunology Research Laboratory, Ely, UK) and diluted 1:25 in 100 µL FACS buffer. All stained cells were then washed and resuspended in 200 µL FACS buffer and run on a BD FACS Canto II. Analysis was performed using FACS Diva software v 8.0.

### 4.4. Osteogenic and Adipogenic Differentiation Potential of Mesenchymal Stem Cells

This was performed as described previously in [55] but with a few minor differences. NT and PTC MSCs were checked for differentiation potential at P3. For Osteogenic differentiation, all cells in this study were plated into 12 well plates at 2.5 × 10^5^/cm^3^ in MSC media for 2 days and then transferred into osteogenic media (StemPro, Gibco, Paisley, UK) and the cultures were maintained for 21 days in 5%CO_2_ and 5%O_2_. For adipogenesis, cells were plated as above for 2 days in MSC media and then transferred into adipogenic differentiation media (StemPro, Gibco, Paisley, UK) and cultured for 21 days. Histological evaluation for osteogenic differentiations involved staining cells with Alizarin red (2% wt/vol) alizarin red (pH 4.3 with 10% [vol/vol] ammonium hydroxide) for 2 h at room temperature before being washed with phosphate-buffered saline. For determination of adipogneic differentiation, cells were stained with Oil Red O (stock solution of 30% [vol/vol] oil red O in isopropanol diluted to 60% (vol/vol) in ddH2O) for 5 min at room temperature and then rinsed with tap water until clear.

### 4.5. Semi–Quantitative PCR Analysis of Differentiated MSCs

RNA was isolated from both MSC populations after differentiation (see above) using the RNeasy micro plus kit (Qiagen, Manchester, UK) and cDNA synthesized using the Tetro-cDNA synthesis kit (Bioline, Nottingham, UK), respectively, as per manufacturer’s instructions. PCR was performed as described previously [54]. Primers used in this study included *aP2* F: 5′-GCCTTCTCCATGGTGGTGGTGAA-3’, R: 5′-GCACCGTCAAGGCTGAGAAC-3′; *ALP* F: 5′-AACCCCAAGATGCACAACTC-3′, R: 5′-GCTTAGCCTCGTCGATGAA-3′; *BSP* F: 5′-AGTGAGAGAGGCAACCTGGAGA-3′, R: 5′-ACACTCGGACCACATCCTTC-3′; and *GAPDH* F: 5′-GCCTTCTCCATGGTGGTGGTGAA-3′, R: 5′-GCACCGTCAAGGCTGAGAAC-3′, which was used as a loading control.

### 4.6. Single Cell RNA Sequencing

All scRNA seq was performed in collaboration with the Genomics Core Facility, Newcastle University. NT and PTC MSCs were cultured and harvested at P3. Cells were suspended in complete MGPM (the media were filtered prior to use through a 30-mm syringe filter) at 100 cells/µL. The cells were then loaded onto a Chromium Single Cell B Chip (Chromium Chip B Single Cell Kit 16rxns 1000074) before being run simultaneously. Single cell RNA sequencing libraries were constructed using a Chromium Single Cell 3′ Library & Gel Bead Kit v3 (16 kit 1000075) and Chromium i7 Multiplex Kit (96 rxns PN-120262). GEMs were generated, cDNA barcoded, GEMs cleaned, and cDNA amplified as per the manufacturer’s protocol (User Guide: Chromium Single Cell 3′ Reagents Kits v3).

cDNA generated using the 10x Genomics chromium single cell workflow was analyzed using a TapeStation 4200 system with a high sensitivity D5000 ScreenTape assay (Agilent, Santa Clara, CA, USA) to assess cDNA concentration according to the manufacturer’s instructions. In brief, all reagents were left to equilibrate to room temperature and vortex mixed. The ladder was prepared by adding 2 µL High Sensitivity D5000 sample buffer to 2 µL ladder. In total, 2 µL High sensitivity D5000 buffer was added to 2 µL cDNA sample, then the sample was vortexed at 2000 rpm for 1 min and loaded on to the TapeStation and analyzed using Tapestation analysis software v5.1. The 3′ gene expression library was performed as per the manufacturer’s protocol (User Guide: Chromium Single Cell 3′ Reagents Kits v3). The constructed libraries were sequenced on an Illumina NovaSeq 6000 System at the Genomics Core Facility, Newcastle University, with a sequencing depth of 13,000–90,000 reads per cell.

### 4.7. Bioinformatics Analysis

Single cell RNA sequencing data were received from the Genomics Core Facility (Newcastle University) in the form of Illumina base call (BCL) files. Using the Cell Ranger (Version 3.0.1, 10xGenomics), workflow BCL files were demultiplexed and converted to FASTQ files using the Cell Ranger mkfastq pipeline. Cell Ranger count was then used to perform the alignment to the human reference genome GRCh38 and for filtering, barcode, and unique molecular identifier (UMI) counting.

Filtered Cell Ranger count data were then further processed using R v4.2.2 [56] and Seurat (version 4.4). Seurat objects were created for each dataset. Quality control was performed to exclude dead cells, debris, low quality reads and empty droplets from downstream analysis. Filtering was applied to both MSC populations to ensure only cells were retained that had a minimum of 1000 counts, a minimum of 500 genes and a mitochondrial gene percentage of 20%. The DoubletFinder package v2.0.4 was used as a predictive tool to remove likely cell doublets from the datasets.

### 4.8. Statistical Analysis for Single Cell RNA Sequencing

Seurat objects for the four datasets were down-sampled to 4000 cells and merged in two phases, firstly the NT and PTC populations and then the full datasets. Merged datasets were integrated and batch corrected using Harmony (version 1.0.3). Clusters of cells were identified, visualized, and statistically analyzed using Seurat. Non-parametric Wilcoxon rank sum tests were used to determine differential expression between genes in the NT MSC and PTC MSC datasets via the FindAllMarker and FindMarkers methods implemented in Seurat.

### 4.9. Statistical Analysis of 28 Genes Identified as Differentially Expressed Genes Upregulated in PTC MSCs

The 28 genes identified as differentially expressed and upregulated in the PTC MSCs in our single cell RNA-sequencing analysis were compared using datasets from 32 cancer studies included in the TCGA Pan Cancer Atlas combined study available via cBio Cancer Genomics portal. This is an open access resource http://cbioportal.org accessed on 2 April 2025) that allows for exploration of multidimensional cancer genomics datasets. Analysis was based on 10,967 samples and the inclusion of RNA-sequencing data; outcomes were based on overall survival and the logrank test *p* value. The results shown in Supplemental Figure S2 were generated in part by cBio Cancer Genomics portal [57,58,59].

## 5. Conclusions

We believe our study is the first to show annotation of gene expression within subpopulations of patient-tissue derived NT and PTC MSCs that points to the similarities in MSCs, whilst also showing differential expression of several genes that support a role for PTC MSCs in PTC progression. Moreover, our data shows that PTC MSCs express several members of the *S100A* and *IGFBP* families. S100A10, S100A6, S100A4 and IGFBP3 have all been linked to PTC with lymph node metastasis (LNM) [42,44,55]. Targeting these genes in PTC MSCs may therefore have potential therapeutic value in treating PTC with LNM. In addition, our analysis identified what may be a novel subpopulation of cells within both MSC populations. This subpopulation of MSCs expresses stromal markers, but also expresses the transcription markers PAX8, FOXE1 and NKX2.5, all of which are involved in thyroid gland development. Cells expressing these transcription factors have been reported to give rise to thyroid-hormone-producing follicular cells [60]. This might suggest a role for this subpopulation in thyroid growth in both normal and pathological conditions. This subpopulation of NT MSCs could possibly be employed as a future cellular therapy for use in regeneration of injured thyroid glands. Future direction for this work will include increasing the number and types of malignant thyroid tissues for further transcriptional analysis and determining functionality of both NT and PTC MSCs.

## Figures and Tables

**Table 1:** Single cell RNA sequencing data analysis showed 28 differentially expressed genes in PTC vs. NT MSCs that have been reported to be involved in cancer progression. Any 0 values indicate *p* value < 2.225074 × 10^−308^.

Gene	*p* Value	Average Log2FC	PCT%	NT%	*p* Value Adjusted (Bonferroni Correction)	MSC Population
IGFBP7	3.0152 × 10^−30^	0.18022276	0.985	0.988	5.0513 × 10^−26^	PTC
IGFBP3	0	1.568476	0.767	0.309	0	PTC
IGFBP5	1.65 × 10^−74^	0.573006	0.775	0.647	2.77 × 10^−70^	PTC
IGFBP6	1.67 × 10^−218^	0.586049	0.987	0.953	2.79 × 10^−214^	PTC
S100A4	2.81 × 10^−91^	0.439812	0.572	0.441	4.71 × 10^−87^	PTC
S100A6	4.73 × 10^−62^	0.255939	1	1	7.92 × 10^−58^	PTC
S100A10	0	0.550230	1	0.992	0	PTC
PRRX1	1.20 × 10^−42^	0.383299	0.75	0.64	2.01 × 10^−138^	PTC
PCOLCE2	0	0.609609	0.42	0.14	0	PTC
HIF1A	0	0.496238	0.941	0.864	0	PTC
SH3BGRL3	4.03 × 10^−280^	0.460636	0.999	0.999	6.75 × 10^−276^	PTC
WWTR1	0	0.676019	0.718	0.512	0	PTC
CDH6	0	0.724795	0.509	0.153	0	PTC
FBN1	0	0.790647	0.986	0.893	0	PTC
HEG1	4.92 × 10^−147^	0.389943	0.466	0.288	8.24 × 10^−143^	PTC
CST1	6.17 × 10^−147^	0.438862	0.144	0.03	1.03 × 10^−142^	PTC
MAZ	2.67 × 10^−169^	0.392678	0.518	0.33	4.48 × 10^−165^	PTC
PGK1	0	0.569080	0.954	0.867	0	PTC
SPOCK1	1.30 × 10^−186^	0.399679	0.967	0.915	2.17 × 10^−182^	PTC
MARCKSL1	1.12 × 10^−303^	0.541839	0.722	0.494	1.88 × 10^−299^	PTC
LTBP1	2.12 × 10^−216^	0.457493	0.921	0.845	3.55 × 10^−212^	PTC
GNAS	0	0.793568	0.969	0.851	0	PTC
NR2F2	2.55 × 10^−170^	0.448546	0.819	0.716	4.28 × 10^−166^	PTC
PLS3	4.07 × 10^−271^	0.490727	0.755	0.577	6.81 × 10^−267^	PTC
EDIL3	0	0.590966	0.463	0.15	0	PTC
SPARC	0	0.944666	0.994	0.881	0	PTC
SCUBE3	1.51 × 10^−267^	0.474113	0.292	0.08	2.53 × 10^−263^	PTC
SOX4	0	0.721343	0.843	0.624	0	PTC

## Data Availability

The data presented in this study are available on request from the corresponding author.

## Supplementary Materials

The following supporting information can be downloaded at: https://www.mdpi.com/article/10.3390/ijms26104957/s1.
