# Supplementary material for: Single Cell RNA Sequencing of Papillary Cancer Mesenchymal Stem/Stromal Cells Reveals a Transcriptional Profile That Supports a Role for These Cells in Cancer Progression
Source: Int J Mol Sci. 2025 May 21;26(10):4957. doi: 10.3390/ijms26104957 (PMC12112585; doi:10.3390/ijms26104957)
Supplement: Supplementary file 1 [file ijms-26-04957-s001.zip › ijms-3596866-supplementary.pdf]

Supplementary Table S1. Percentage of MSCs expressing vascular and stromal cell genes and median values of these expressions.

Gene name	% of NT MSCs expressing gene	Median expression in NT MSCs	% of PTC MSCs expressing gene	Median expression in PTC MScs
VEGFA	88.4375	1.4	66.275	1.4
ANGPT1	15.3	0.6	7.5625	0.8
ANGP2	2.4625	0.6	2.3625	0.9
THY1	50.65	1	81.1125	1.5
CD44	91.7125	1.4	78.5875	1.4
MCAM	2.5	0.7	0.9	0.9
ALCAM	60.9875	0.9	53.75	1.2
ENG	68.0625	1	72.9875	1.4

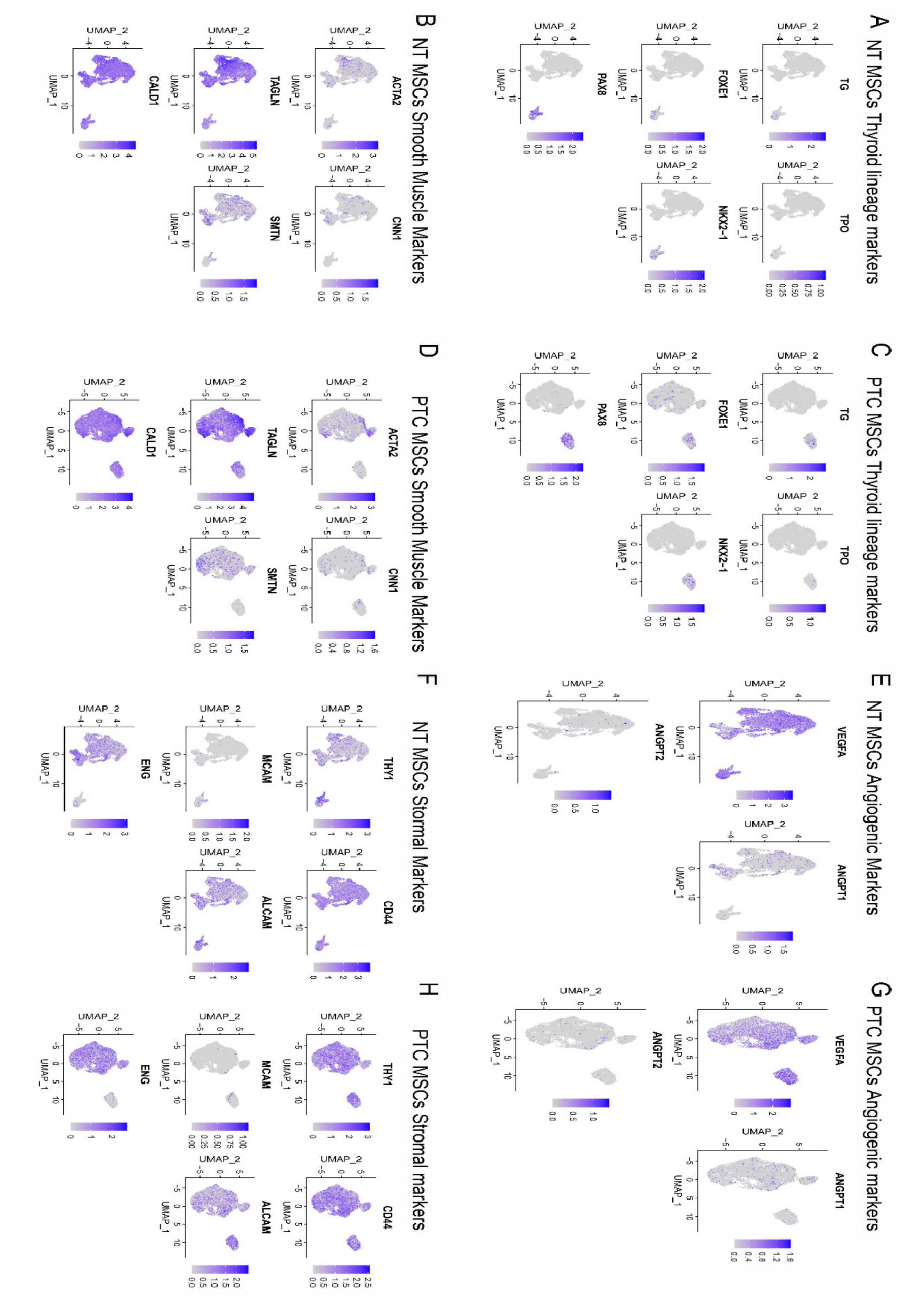

Supplementary Figure S1. UMAPs showing expression patterns of data represented by violin plots in Figure 2.

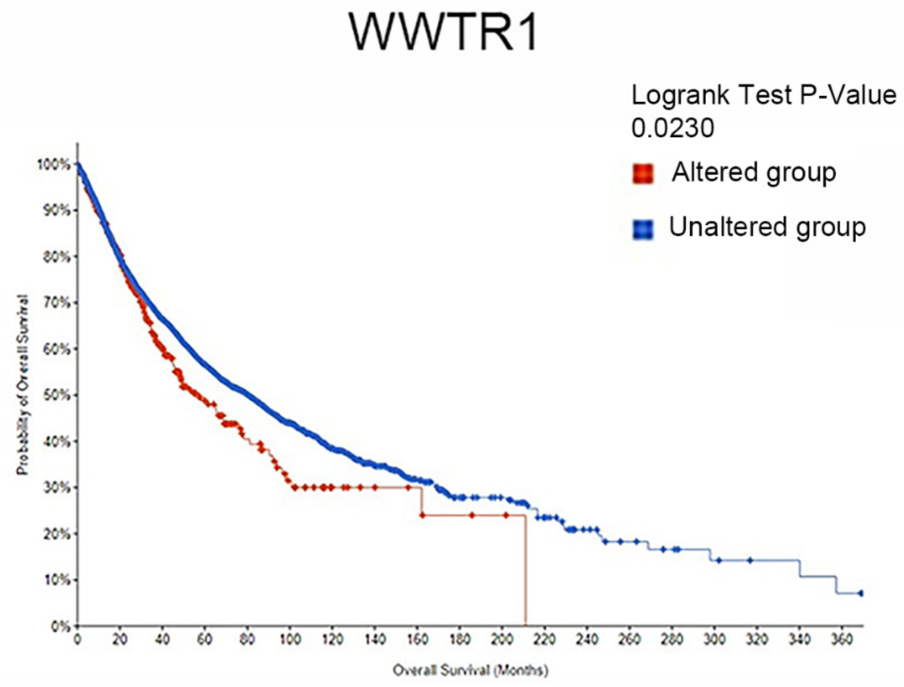

Supplementary Figure S2. Alterations in genetic expression of WWTR1 results in reduced overall survival rate (data from cBIO Cancer Genomics Portal).
